# Peptidomic Identification of Behaviour-Modulating Putative Neuropeptides in *Schistosoma mansoni* Miracidia

**DOI:** 10.3390/ijms27062839

**Published:** 2026-03-20

**Authors:** Conor E. Fogarty, Saowaros Suwansa-ard, Tomas Lang, Phong Phan, Mary G. Duke, Russell C. Wyeth, Scott F. Cummins, Tianfang Wang

**Affiliations:** 1 Centre for Bioinnovation, University of the Sunshine Coast, Maroochydore, QLD 4558, Australia; cfogarty@usc.edu.au (C.E.F.); ssuwansa@usc.edu.au (S.S.-a.); tlang1@usc.edu.au (T.L.); phantuanphong1986@gmail.com (P.P.); scummins@usc.edu.au (S.F.C.); 2 School of Science, Technology and Engineering, University of the Sunshine Coast, Maroochydore, QLD 4558, Australia; 3 QIMR Berghofer Medical Research Institute, Brisbane, QLD 4006, Australia; mary.duke@qimrb.edu.au; 4 Department of Biology, St. Francis Xavier University, Antigonish, NS B2G2W5, Canada; rwyeth@stfx.ca

**Keywords:** schistosomiasis, *Schistosoma mansoni*, miracidia, putative neuropeptide, behavioural bioassay, parasite-host interaction

## Abstract

Neuropeptides regulate diverse physiological and behavioural processes in parasites, yet their functional roles in the infective larval stages of *Schistosoma mansoni* remain poorly defined. In this study, we identified miracidia-derived putative neuropeptides and examined their roles in regulating miracidial behaviour. Peptidomic analysis revealed ten putative neuropeptides, including five whose proteomic identification in this life stage was previously unreported. Neuropeptide precursor proteins were evaluated for stage-specific expression and *Schistosoma* genus specificity to prioritise candidates with potential functional and biocontrol relevance. Protein–protein interaction analysis identified Smp_176700 as a highly connected neuropeptide precursor associated with proteins implicated in miracidial structure and infection. Eight putative neuropeptides derived from six precursor proteins were synthesised and externally applied to miracidia in acute (1 min) and prolonged (360 min) behavioural assays. During acute exposure, most peptides induced significant concentration-dependent behavioural changes at 3 mg/mL and 0.1 mg/mL, characterised by reduced swimming velocity and increased directional change, with no significant effects at 0.01 mg/mL. Prolonged exposure revealed peptide-specific effects, with ASLSYF-OH and FLLGLPPSLRQH-OH producing the most pronounced behavioural modulation. These findings demonstrate that *S. mansoni* miracidia express bioactive neuropeptides capable of modulating larval behaviour, providing insight into schistosome neurobiology and identifying potential targets for transmission-blocking interventions.

## 1. Introduction

Schistosomiasis is one of the deadliest but neglected tropical diseases in the developing world. The disease is caused by infection from *Schistosoma* digenetic trematodes such as *Schistosoma mansoni*. It is one of the most common and consequential cofactors of HIV [1,2]. Furthermore, schistosomiasis is a common comorbidity with anaemia and causes complications in the gastrointestinal, urogenital, and cardiopulmonary systems [3,4]. Improvements in infrastructure, including more comprehensive and readily available education and water supply systems, have been shown to decrease the spread of human schistosomiasis [5,6]. However, chemical interventions, such as interfering with the infection processes of *Schistosoma* miracidia (which infect intermediate molluscan hosts) and cercariae (which infect definitive mammalian hosts), are also promising approaches to minimise the spread of schistosomiasis [7,8,9,10,11,12,13].

Interference with *Schistosoma* neurobiology has been considered as a targeted approach to manage schistosomiasis [14], which is assisted by the comprehensive characterisation of the neurochemistry of *S. mansoni* at mature and intramammalian stages [15,16]. Following the publication of the *S. mansoni* genome, 16 different neuropeptide families were identified [17,18], and since then, 45 neuropeptides have been identified [19]. The most abundant group comprises neuropeptides F (NPF) peptides, which are homologous to vertebrate neuropeptides Y (NPY) peptides but are largely restricted to the Platyhelminth phylum (“F” and “Y” denote the conserved C-terminal residues phenylalanine and tyrosine, respectively) [17,20]. Enzymes involved in neuropeptide amidation have been considered ideal pharmaceutical targets because of their essential role in parasite movement and high species specificity, minimising the risk of biocontrols harming non-target species [21,22].

Fewer neuropeptide targets have been identified in the *S. mansoni* infective stages than in the mature intramammalian stage. Yet neuropeptidergic activity is essential for miracidial and cercarial locomotion, leading to host identification. Thus, neuropeptides are promising targets for biocontrols [23]. The effectiveness of neurochemical antagonism has been demonstrated in cercariae, whereby a suspected serotonin antagonist was lethal to cercariae within 3 min post-exposure at concentrations as low as 25 nM [24]. However, because serotonin is a ubiquitous neurotransmitter, manipulation with a serotonin antagonist would threaten non-target species. Therefore, identification of species-specific neuropeptide targets is necessary to minimise this risk.

Of the 17 putative guanine protein-coupled receptors (GPCRs) identified in *S. mansoni* miracidia, three were annotated as neuropeptide-like receptors [25]. Several recent experiments provide templates for examining the effects of neuropeptides on behaviour. For instance, one study externally applied *S. mansoni* cercariae-derived neuropeptides to cercariae and noted various behaviour changes, including increases in spinning, directional change, and head-pulling [8]. These results implicated specific putative neuropeptides in regulating behaviour changes related to host identification and infection. Therefore, these neuropeptides may function as viable species-specific targets. The viability of neuropeptide targeting was further illustrated in a study on the soybean cyst nematode *Heterodera glycines*, which observed that neuropeptide exposure increased infectivity, while knockdown of genes that encoded the neuropeptide precursor proteins decreased infectivity [26]. Conducting similar behaviour bioassays on neuropeptides derived from *S. mansoni* miracidia could identify putative biocontrol targets and further elucidate the host-infection mechanism.

In this study, we identify and functionally characterise miracidia-derived putative neuropeptides from *S. mansoni* using integrated peptidomic and in silico analyses combined with quantitative behavioural bioassays. Candidate neuropeptides were prioritised based on stage-specific expression and *Schistosoma* specificity, informed by a miracidia transcriptomic database. The effects of selected neuropeptides on miracidial behaviour were evaluated under acute (1 min) and prolonged (360 min) exposure conditions. By linking defined neuropeptides to measurable behavioural modulation, this study advances understanding of schistosome larval neurobiology and provides a foundation for the development of species-specific neuroactive strategies to interrupt parasite transmission.

## 2. Results

### 2.1. Putative Neuropeptide Precursors Identified in Miracidia

A total of 13 proteins were identified from the miracidia protein extracts, supported by 14 peptides with high-confidence MS/MS spectra (Table S1). The BlastP analysis confirmed the presence of four putative neuropeptide precursor proteins [Smp_142160 (Sm-npp-17), Smp_004710 (Sm-npp-30), Smp_201600 (Sm-npp-36), and Smp_200440 (Sm-npp-40)] previously identified in *S. mansoni* adults and cercariae [16,17]. The other nine putative neuropeptide precursors have been identified in chromosomal studies aiming to elucidate sex differences in the parasite [27]. This is the first time their presence has been confirmed in the miracidia stage through proteomic analysis. Novel proteins Smp_325550, Smp_325560, and Smp_325580 were excluded from consideration as neuropeptide precursors due to the absence of predicted signal peptides. Smp_321760 and Smp_346020 lacked predicted mature neuropeptides (predicted to be cleaved at monobasic/dibasic/tribasic sites by NeuroPred), hence these proteins were also excluded from consideration (Table S2).

**Figure 1:**
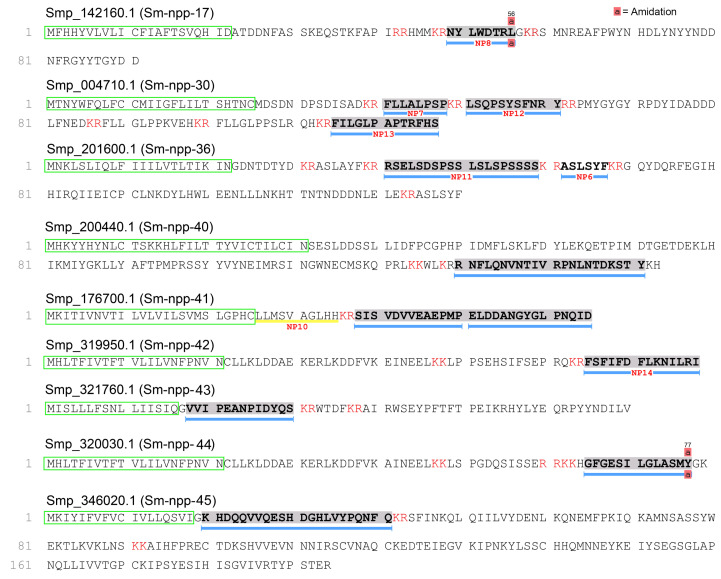
Identification of neuropeptide precursors in the miracidia stage of *S. mansoni*. The peptide segments, supported by high-confidence MS/MS spectra (supported by at least five consecutive *b* or *y* ions), are displayed in grey shade and underlined with blue bars. A yellow underline was also chosen for peptide synthesis. Predicted signal peptides are shown in a green frame, and cleavage sites are in red font.

There were 23 putative neuropeptides identified from cleavage sites predicted by NeuroPred from the confirmed precursor proteins, of which a total of 8 were supported by LC-MS/MS spectra. These included NYLWDTRL-NH_2_ (Sm-npp-17), FILGLPAPTRFHS-OH (Sm-npp-30), RSELSDSPSSLSLSPSSSS-OH (Sm-npp-36), RNFLQNVNTIVRPNLNTDKSTY-OH (Sm-npp-40), HGFGESILGLASMY-NH_2_ (Smp_320030), RGFGESILGLASMY-NH_2_ (Smp_320000), VVIPEANPIDYQS-OH (Smp_321760), FSFIFDFLKNILRI-OH (Smp_319950). A further five and four putative neuropeptides were predicted from Sm-npp-30 and Sm-npp-36, respectively (Figure 1 and Table S2). A total of four putative neuropeptides were derived from previously unconfirmed neuropeptide precursor proteins, including LLMSVAGLHH from Smp_176700.1 (Sm-npp-41) and LLKLDDAEKERLKDDFVKEINEEL-OH, LPPSEHSIFSEPRQ-OH, and FSFIFDFLKNILRI-OH from Smp_319950.1 (Sm-npp-42). These putative neuropeptides were respectively labelled Sm-npp-41 and Sm-npp-42 to stay consistent with the nomenclature used to describe *S. mansoni* neuropeptide precursor proteins. Also identified were putative neuropeptide precursor proteins Smp_320000 and Smp_320030, which each contained three predicted neuropeptides.

### 2.2. PPI Analysis Identified Interaction Between Putative Neuropeptides and Structural Proteins

**Figure 2:**
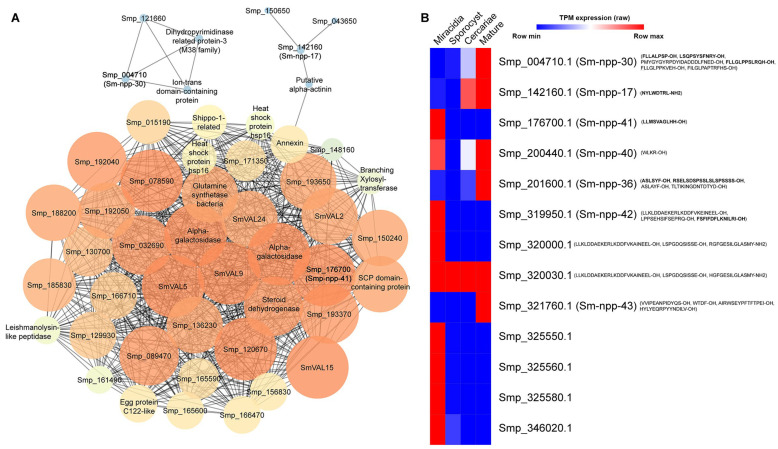
PPI and gene expression analysis of identified *S. mansoni* miracidia proteins. (**A**) PPI analysis (confidence score above 0.15), with putative neuropeptide precursors in bold. For colour coding: nodes are on a spectrum from high degree (red) to low degree (blue). The size of the circles also corresponds to the node degree. (**B**) The relative gene expression of all identified proteins, including precursors of putative neuropeptides, at different stages: miracidia, sporocyst, cercariae, and mature schistosome. This was constructed from a previous report; some proteins were not included [28]. The corresponding gene IDs of the current version of the *S. mansoni* genome are shown after “/”. The sequences of predicted putative neuropeptides are shown in parentheses. (Expression values in transcripts per million units, TPMs, are shown in Table S4).

To identify potential interactions between putative neuropeptides and other proteins in miracidia, a PPI analysis using the entire proteome of *S. mansoni* miracidia. The PPI network analysis encompassed all annotated proteins, including neuropeptide precursors (Figure 2A). Most edges demonstrated an insignificant level of interplay, likely due to the uncharacterised status of almost 76.9% of identified proteins. In this network, SmVal15 emerged as the most interconnected node, with links to various proteins, including Smp_009810, Smp_015190, Smp_021460, Smp_032690, Smp_048050, Smp_049230, as well as several other structural proteins (Table S3). Further statistical analysis of the PPI network is shown in Figure S1. A majority of the PPIs had a path length of 1, meaning the nodes were intensely connected. This observation is consistent with the high degrees of most nodes, which exceeded 37. While the highest betweenness and closeness centrality values were 0.17 and 1.0, respectively, most neighbouring nodes exhibited relatively lower centrality values.

Among the putative neuropeptide precursors, only Smp_004710, Smp_142160, and Smp_176700 showed predicted interactions with other proteins. The PPI network did not predict any direct interaction between these putative neuropeptide precursors. Instead, neuropeptide precursor proteins Smp_004710 and Smp_121660, dihydropyrimidinase-related protein-3 (M38 family), and ion-trans domain-containing protein were predicted to form a cluster of interactions separate from the other proteins. Additionally, Smp_142160 was predicted to interact with Smp_150650, Smp_043650, and putative alpha-actinin. This alpha-actinin, in turn, was linked to the larger protein cluster via annexin (Figure 2A). In contrast, Smp_176700 exhibited the broadest range of predicted interactions, including various venom allergen-like, alpha-galactosidase, leishmanolysin-like peptidase, annexin, steroid dehydrogenase, and a wide range of other established and novel proteins. Some of these proteins, such as annexin and leishmanolysin-like peptidase, have been strongly implicated in infection and immunosuppression. Therefore, it can be predicted that among the putative neuropeptide precursors, Smp_176700 may have a significant role in regulating miracidia behaviour. Nonetheless, it is advisable to conduct further analysis on the other putative neuropeptide precursors to strengthen protein–protein interaction analysis.

The gene expression levels corresponding to identified neuropeptide precursor proteins were derived from a previous study, which compared the transcriptomes of different stages of *S. mansoni*, including miracidia, sporocysts, cercariae, and adult schistosomes [28]. The relative expression of protein nodes and precursor proteins of putative neuropeptides is compared in Figure 2B. Most of the genes had relatively low levels of expression in miracidia (Table S4).

### 2.3. Analysis of Putative Neuropeptides on Schistosoma mansoni Miracidia Behaviour

A total of 8 putative neuropeptides were chosen for assessment of bioactivity (Table 1). The chosen putative neuropeptides were derived from putative precursor proteins with high conservation among *Schistosoma* and with expression detected in the miracidia stage. These neuropeptide precursor proteins also showed either no homologues outside of the *Schistosoma* genus or only one homologue in closely related *Trichobilharzia* (File S1). Among these precursor proteins, those with the highest relative expression in miracidia included Smp_176700.1 and Smp_201600.1 (Figure 2B and Table S4). In contrast, Smp_320000 and Smp_320030 were excluded due to low expression in the miracidia stage. Furthermore, the selected putative neuropeptides were required to be relatively small (<2 kDa) as this would ensure their ability to effectively penetrate miracidia; however, they also needed to contain a minimum of 5 amino acids to determine functional specificity. This requirement excluded putative neuropeptides derived from Smp_200440 and Smp_346020. Therefore, the final selection comprised six putative neuropeptides, including four derived from established neuropeptide precursor proteins: NP7, NP12 and NP13 (Sm-npp-30); NP8 (Sm-npp-17) and NP6 and NP11 (Sm-npp-36). Additionally, two putative neuropeptides were derived from novel neuropeptide precursor proteins: NP10 (Smp_176700.1) and NP14 (Smp_319950.1).

**Figure 3:**
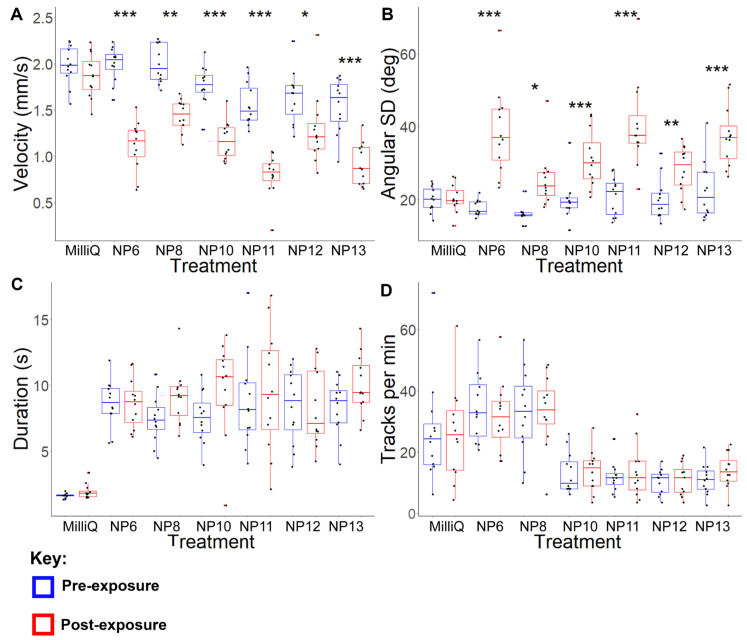
Changes in *S. mansoni* miracidial behaviour from one min pre-exposure and post-exposure to Milli-Q water and 3 mg/mL NP6, NP8, NP10, NP11, NP12, NP13. (**A**) Average velocity (mm/s); (**B**) Angular SD (degrees); (**C**) duration of presence (s); (**D**) miracidia per min. A box plot indicates median, 25th and 75th percentiles, minimum and maximum data, with outliers represented by dots. Colour codes: Blue represents pre-exposure and red represents post-exposure measurements. Pre-exposure values (blue) correspond to baseline behaviour for each treatment group; variation between groups reflects biological differences among independent batches of miracidia. A two-way ART-ANOVA test was used to calculate *p*-values for the mixed-effects interaction of pre-exposure and post-exposure neuropeptide treatments against Milli-Q water: * *p* < 0.05, ** *p* < 0.01, and *** *p* < 0.001.

Acute behavioural bioassays were conducted to observe miracidia behaviour changes over one min post-exposure to putative neuropeptides at 3 mg/mL (Figure 3), 0.1 mg/mL (Figure S2), and 0.01 mg/mL (Figure S3). Due to excess hydrophobicity, NP14 was excluded from the behaviour bioassay analysis following preliminary experiments, as our previous study revealed that highly hydrophobic peptides cause the Milli-Q water on the glass slide to dissipate [8]. Additionally, NP7 was excluded from further bioassays as it failed to induce any significant behavioural changes, even at the highest concentration of 3 mg/mL. All other putative neuropeptides induced similar significant changes at 3 mg/mL and 0.1 mg/mL (Table S5). These behavioural changes included significant reductions in velocity and significant increases in angular SD relative to the control at 3 mg/mL and significant increases in angular SD at 0.1 mg/mL (Table S6). The similarity in the effects induced by exposure to all putative neuropeptides suggests these findings could be evidence of non-specific responses to high peptide concentrations. In contrast, at a concentration of 0.01 mg/mL, there were no significant differences in any behavioural metrics for any putative neuropeptides. Therefore, prolonged behavioural bioassays were conducted at this concentration, resulting in incubation at a final concentration of 157 ng/mL.

**Figure 4:**
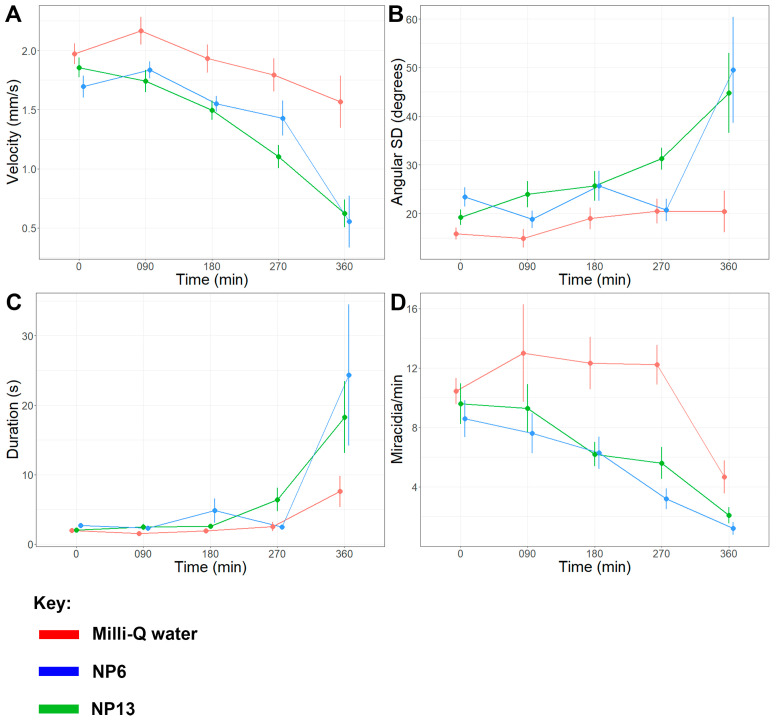
Changes in *S. mansoni* miracidia behaviour pre-exposure and 90 min, 180 min, 270 min, and 360 min post-exposure to Milli-Q water and 0.01 mg/mL NP6 and NP13. (**A**) Average velocity (mm/s); (**B**) Angular SD (degrees); (**C**) Duration of presence (s); (**D**) Miracidia per min. A two-way ANOVA test was used to calculate *p*-values for the mixed-effects interaction of pre-addition and 90 min, 180 min, 270 min, and 360 min post-addition neuropeptide treatments against Milli-Q water. Colour: Red: Milli-Q; Blue: NP6; Dark Green: NP13.

The prolonged exposure analysis initially revealed few significant changes within the first 180 min post-exposure (Table 2 and Table S7). However, at 270 min and 360 min post-exposure, NP6-exposed miracidia displayed several significant changes in multiple behaviour metrics indicative of increased miracidia lethargy relative to the control (Movies S1 and S2). The observed alterations included a progressive reduction in average velocity, a decrease in tracks per minute, and an increase in the average duration of presence over the 360 min observation period. Compared against pre-exposure, exposure to NP6 caused a significant decrease in average velocity of 40.62% at 270 min (*p* = 0.0022) and 66.50% at 360 min (*p* = 0.0006) post-exposure (Figure 4A). NP6-exposed miracidia also presented a significant increase in angular SD of 133.04% at 360 min post-exposure (*p* = 0.0071) (Figure 4B) and a significant increase in average duration of presence of 211.46% at 270 min (*p* = 0.0238) and 788.19% at 360 min (*p* = 0.0009) post-exposure (Figure 4C). Lastly, the putative neuropeptide also caused average tracks per min to significantly decrease by 35.42% at 180 min (*p* = 0.0276) and 41.67% at 270 min (*p* = 0.0094) post-exposure (Figure 4D). These results collectively suggest that NP6 exposure caused miracidia to exhibit consistently slower and less linear movements, contributing to a decrease in the number of miracidia in the field of view (FOV).

Another putative neuropeptide, NP13, affected the same behaviour metrics as NP6; however, only significantly at 360 min post-exposure (Movie S3). Comparing miracidia pre-exposure to 360 min post-exposure, NP13 induced a significant 67.36% decrease in average velocity (*p* = 0.0193), an 801.28% significant increase in average duration of presence (*p* = 0.0008), and a 111.56% significant increase in angular SD (*p* = 0.0389). However, significant decreases in tracks per min were only observed at 270 min post-exposure. These behavioural changes suggest that NP13 exposure causes a similar increase in slow and circular movements to NP6; however, a longer duration of exposure is necessary for the change to be significant.

No other putative neuropeptides caused significant changes in all metrics relating to miracidial behaviour in the FOV. In contrast to NP6 and NP13, NP12 primarily affected the number of miracidia detected in the FOV, without inducing consistent changes in velocity, angular SD, or duration of presence. NP11 caused the earliest and most consistent decreases in tracks per min, suggesting higher miracidial sensitivity to this putative neuropeptide, and possibly a lethal effect. Following exposure to NP11, tracks per min significantly decreased by 50.49% at 90 min (*p* = 0.0053), 48.75% at 180 min (*p* = 0.0129), and 79.15% at 270 min (*p* = 0.0000) post-exposure. Exposure to NP12 induced similar significant decreases in tracks per min, including decreases of 74.00% at 180 min (*p* = 0.0183) and 80.00% at 270 min (*p* = 0.0247) post-exposure, but no significant effects were detected at earlier intervals. The absence of concurrent changes in other behavioural metrics suggests that NP12 may reduce miracidial presence in the FOV through progressive loss of motility or viability rather than inducing overt behavioural dysregulation.

From this data, it appears that NP6 and NP13 are likely bioactive, causing significant behaviour changes in several behaviour metrics, while NP11 and NP12 predominantly reduce the quantity of miracidia observed in the FOV, consistent with potential lethal or strongly inhibitory effects. In contrast, NP8 and NP10 exposure only produced significant decreases in tracks per min at individual time-points (180 min and 360 min post-exposure, respectively), suggesting relatively low bioactivity to miracidia.

### 2.4. Structural Analysis of Bioactive Putative Neuropeptides That Modify Schistosoma mansoni Miracidia Behaviour

**Figure 5:**
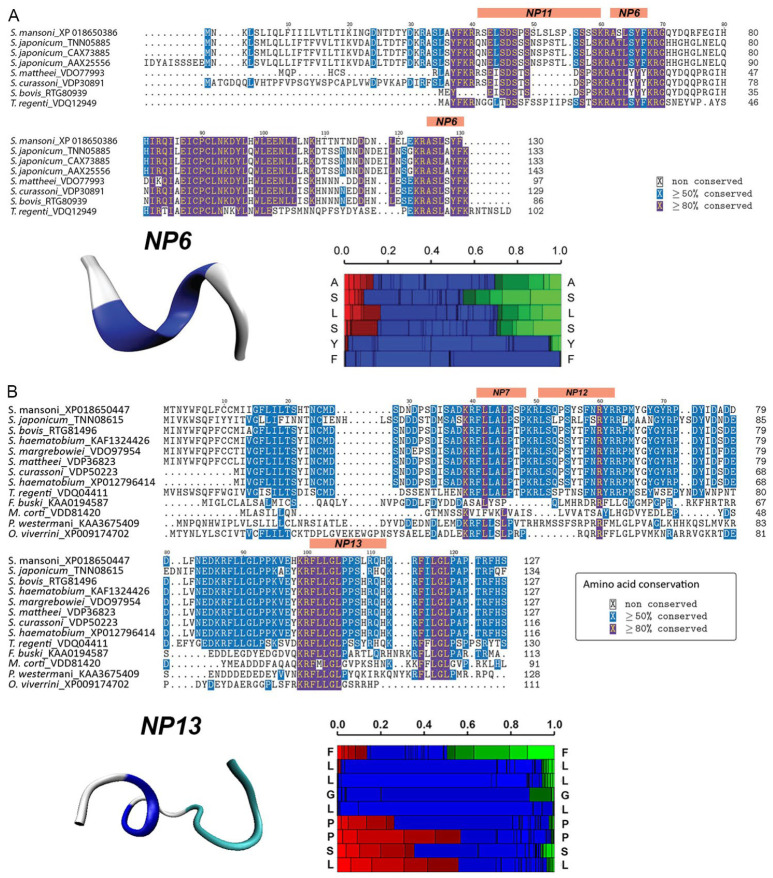
Structure of bioactive peptides and comparative sequence analysis of their respective precursor proteins, including information on conserved amino acids. (**A**) Sm-npp-36 and NP6 (ASLSYF-OH); (**B**) Sm-npp-30 and NP13 (FLLGLPPSLRQH-OH). Multiple sequence alignment of Sm-npp-33. Structural Alphabet (SA) (vertical axis) at each position. Colour code: red: helical, green: extended, blue: coil. Heatmaps and structural models were generated using PEP-FOLD3.

The significant and consistent bioactivity observed in miracidia following exposure to NP6 and NP13 prompted a more detailed peptidomic analysis. NP6 exhibited a high degree of conservation within the *Schistosoma* genus, with limited homology in other species, except for the closely related *Trichobilharzia*, which implies a high level of functional specificity to these trematodes (Figure 5A). Additionally, NP6 displayed a notable inclination towards turn helices, exhibited pH-neutrality, and moderate hydrophobicity. On the other hand, NP13 exhibited a similarly high level of conservation within *Schistosoma*, with minimal homology in other trematodes, including *Fasciolopsis buski* and *Mesocestoides corti* (Figure 5B). Secondary structure analysis indicated a high propensity for turn helices and an extended structure throughout the whole sequence. Detailed descriptions of the other neuropeptides used in the acute exposure experiments are presented in Figure S4.

## 3. Discussion

This study aimed to identify putative neuropeptides present in *S. mansoni* miracidia and observe their function in behaviour regulation. These goals were achieved through the proteomic analysis of miracidia-derived protein extracts, followed by acute and prolonged exposure behavioural bioassays. The behavioural analysis revealed two putative neuropeptides that induced significant increases in miracidial lethargy and two more that appear to have accelerated miracidial death. These findings provide valuable insights into the potential of neuropeptides as targets for biocontrol strategies aimed at modulating miracidial behaviour. One possible approach could involve the controlled release of synthetic neuropeptides [29,30] or stable analogues in aquatic habitats with high *S. mansoni* prevalence, for example, through baited traps or slow-release matrices designed to either attract miracidia (using peptides that induce host-finding behaviour) or reduce larval survival (using peptides with lethality-associated effects). Such strategies could potentially complement existing environmental control measures. However, practical implementation would require careful evaluation of peptide stability in natural waters, delivery methods that are economically feasible in endemic regions, and potential ecological impacts on non-target organisms. Therefore, further studies assessing environmental persistence, specificity, and safety will be necessary before considering field deployment.

Various studies have investigated the presence and function of neuropeptides in *S. mansoni*. Combining findings from the genomic analysis and a recent investigation into neuropeptides, a total of 30 putative neuropeptides were derived from 11 neuropeptide precursor proteins [16,17]. In this study, that number expanded to 35 with the discovery of an additional five putative neuropeptide precursors identified for the first time in miracidia. All the putative neuropeptide precursors identified in this study from miracidia were supported by high-confidence MS/MS spectra (containing at least five consecutive *b* or *y* ions), high likelihood of signal peptide presence, and predictions of mature peptides. A comparative phylogenetic analysis guided by BLASTp demonstrated a remarkable specificity to the *Schistosoma* genus for all putative neuropeptide precursors. These putative neuropeptides exhibited minimal homology with neuropeptides in other digenetic trematodes, except in the case of the closely related *Trichobilharzia regenti*. The apparent restriction of related sequences to certain schistosome lineages, including neuropathogenic avian schistosomes [31,32], may reflect lineage-specific divergence in neuropeptide signalling systems [33,34]; however, it may also arise from incomplete sampling or annotation of neuropeptide precursor genes in currently available trematode genomic and transcriptomic databases.

The relative gene expression patterns indicated that nine genes were highly expressed in miracidia, strongly implying functional specificity to this developmental stage. Recent studies have also highlighted that eggs and miracidia derived from different anatomical origins (intestinal versus hepatic) can exhibit distinct transcriptional profiles and phenotypic characteristics, indicating additional complexity in early schistosome developmental regulation [35,36]. These observations suggest that neuropeptide expression may already begin during miracidial development within the egg stage, and that future stage-resolved transcriptomic analyses incorporating egg-derived samples will be valuable to clarify the temporal regulation and functional roles of these peptides. In contrast, four encoding genes exhibited higher expression in both cercariae and mature *Schistosoma*, including Sm-npp-30, Sm-npp-17, Sm-npp-36, and Smp_321760. Nonetheless, several putative neuropeptides derived from these precursor proteins were still considered despite the suspected lack of functional specificity to the miracidia stage. Future studies incorporating stage-resolved transcriptomic analyses, including the egg stage, will be important to determine whether these neuropeptides are already expressed during miracidial development prior to hatching or are primarily associated with post-hatching host-seeking behaviour.

In contrast to the acute putative neuropeptide exposure analyses performed on *S. mansoni* cercariae, it was observed that there were negligible differences in miracidia behaviour changes in response to acute exposure to different putative neuropeptides. At concentrations of 0.1 mg/mL and 3 mg/mL, all tested putative neuropeptides, except for NP7, induced significant increases in the magnitude and frequency of directional change. However, none of the putative neuropeptides induced immediate significant behavioural changes at 0.01 mg/mL. Because it is unlikely that all putative neuropeptides regulate the same behavioural responses, it was suspected that the acute exposure provided an insufficient duration to observe behavioural changes beyond generic responses to high peptide concentrations. As a result, prolonged exposure analyses were conducted at 0.01 mg/mL over 360 min to assess longer-term effects of neuropeptide exposure on miracidia behaviour.

In contrast to the acute exposure assay, the prolonged exposure bioassay revealed substantial differences in miracidia responses. After 270 min of exposure to NP6, miracidia revealed significant decreases in average velocity and quantity present, along with significant increases in duration of presence in the FOV and turning magnitude and frequency. These changes in behaviour metrics are consistent with the behaviour of *S. mansoni* miracidia in the vicinity of *B. glabrata* snail-conditioned water [9]. In natural conditions, miracidia typically slow their swimming velocity and alter their trajectory when approaching a compatible snail host. The behavioural changes observed here following NP6 and NP13 exposure, characterised by reduced velocity and increased turning behaviour, may therefore reflect disruption of the coordinated locomotor and sensory processes required for effective host-seeking [37]. Interference with these behavioural programmes would likely impair the ability of miracidia to successfully locate and infect their intermediate host, highlighting the potential of these neuropeptides as transmission-blocking targets. Therefore, the prolonged exposure results implicate NP6 in regulating attraction-like responses. NP13 induced comparable changes in behaviour metrics, although these changes did not become significant until 360 min post-exposure, possibly due in part to its greater molecular weight relative to NP6, which may have impeded its penetration rate. Therefore, these putative neuropeptides should be investigated as potential biocontrol targets that are potentially involved in the locomotion apparatus of host identification [26,38,39]. Another putative neuropeptide of interest was NP11, which induced significant decreases in the number of miracidia present in the FOV at 90 min, 180 min, and 270 min post-exposure. These rapid and consistent decreases in miracidia quantity suggest a lethal effect at 0.01 mg/mL (an effective concentration of 103 nM), indicating high sensitivity to this particular putative neuropeptide.

The behaviour bioassay results from this study showed significant differences compared to those of the *S. mansoni* cercariae putative neuropeptide study. While it is challenging to make a direct comparison between the two studies, as the concentrations of neuropeptides used for miracidia were much lower than those used for cercariae, some interesting contrasts can be noted. In the preliminary acute exposure experiments, miracidia exhibited a weaker response to NP7 exposure compared to other neuropeptides, suggesting relatively low sensitivity to NP7. These behaviour changes contrast with the response induced in cercariae to NP7, which led to significant increases in active state turning magnitude and frequency. Furthermore, NP8 exposure significantly increased the proportion of cercarial passive behaviour throughout the prolonged exposure assay. However, NP8 did not induce consistent and significant changes in miracidia behaviour during prolonged exposure, implying higher cercarial sensitivity to this putative neuropeptide. Surprisingly, acute cercarial exposure to NP6 did not result in a significant change in cercarial behaviour, even though NP6 induced the greatest and most significant changes in miracidia behaviour. One possible explanation for these differences between cercarial and miracidial responses is related to the ciliated plates of miracidia. These structures could potentially impede the interaction between the neuropeptide receptors and putative neuropeptides. Furthermore, the differences in sensitivity to neuropeptides might be attributed to variations in the expression levels of the precursor protein-encoding genes in miracidia and cercariae. For instance, the genes encoding the precursor proteins for NP6 and NP8 were expressed at significantly higher levels in the cercarial stage compared to miracidia (Table S4). The transcriptomic comparison revealed the expression of the NP7 precursor protein encoding gene as over 12 times more highly expressed in cercariae than in miracidia. This could account for the observed differences in responses between these two life stages of the parasite.

The findings related to the putative neuropeptide NP10 are intriguing. Despite the relatively high expression of the NP10 precursor protein encoding gene, the neuropeptide did not induce any significant behavioural responses during the prolonged exposure assay. This suggests that the putative neuropeptide might not be directly involved in miracidia infection behaviour. However, its specificity to miracidia implies a stage-specific function, and it is possible that NP10 plays a role in other aspects of miracidia physiology or behaviour that were not assessed in this study. The protein–protein interaction network implied strongly correlated co-expression between the NP10 precursor protein (Smp-npp-41) and a wide variety of other proteins, including annexin and leishmanolysin-like peptidases. However, the derived peptide NP10 exhibited relatively low bioactivity in the behavioural assays conducted here. This discrepancy may indicate that the precursor protein Smp_176700 performs functions not directly related to the locomotor responses measured in this study, that NP10 may exert biological roles under conditions not captured in our in vitro assays, or that the predictive power of the current PPI network is limited by the large number of uncharacterised proteins in the miracidia proteome. These observations highlight the need for further functional studies to clarify the role of this precursor and its derived peptides. Conversely, there were insufficient predicted interactions to speculate on the underlying chemical mechanisms of NP6, while NP13 was observed to interact with a novel protein, an ion-trans domain-containing protein, and an M38 family protein. Further investigations of the interactions between the NP6 precursor protein and other miracidia proteins should be conducted to elucidate the mechanisms of the NP6 putative neuropeptide.

Future studies should investigate the effects of prolonged exposure to putative neuropeptides not identified in this study. The relative infectivity of miracidia exposed to these neuropeptides identified should be assessed to determine if neuropeptide targeting increases or decreases infectivity, similar to the study on *Heterodera glycines* [26]. These studies should include *Schistosoma*-specific neuropeptides derived from neuropeptide precursor genes identified in miracidia, such as neuropeptide F, RGMIamide, and GFVRIamide [40]. Additionally, it should be confirmed whether the externally applied neuropeptides successfully penetrate the miracidia ciliated plates, or if the behavioural responses are caused by interactions with surface receptors. Further studies using spatial localisation approaches, such as immunostaining or transcript-based in situ analyses, will also be important to determine whether these peptides are associated with neuronal structures within miracidia and to confirm their roles in the schistosome nervous system. Furthermore, miracidia neuropeptides may regulate other essential functions outside of behaviour, including responses to *B. glabrata* immune responses and the miracidia-to-sporocyst transformation. Downregulating the expression of the genes encoding putative neuropeptide precursors and assessing rates of cercarial shedding may confirm the role of these chemicals in miracidia transformation and propagation.

## 4. Materials and Methods

### 4.1. Mouse and Parasite Maintenance Conditions and Ethics Guidelines

*Schistosoma mansoni* (Puerto Rican strain) was maintained under an Australian Department of Agriculture, Fisheries and Forestry Biosecurity permit. All procedures were conducted in strict accordance with animal ethics requirements and approved by the Animal Ethics Committee of the QIMR Berghofer Medical Research Institute, Brisbane (Project No. P3705). *Biomphalaria glabrata* snails (NMRI strain) were maintained in aerated aquaria containing calcium carbonate–conditioned, pH-neutral water at 27 °C under a 12 h light/12 h dark cycle. Snails were fed algae tablets and lettuce, which were thoroughly washed with reverse osmosis water prior to feeding to minimise the introduction of contaminants. Swiss mice used in this study were maintained under Biosecurity Quarantine Control and housed in a quarantine containment area within a Specific Pathogen-Free (SPF) animal facility. All animal procedures were performed in accordance with the recommendations of the Guide for the Care and Use of Laboratory Animals of the National Institutes of Health.

### 4.2. Schistosoma mansoni Miracidia Peptide Extraction Isolation

*Schistosoma mansoni* eggs were collected from infected Swiss mice following euthanasia by CO_2_ inhalation. Mouse livers were perfused with room-temperature 1× phosphate-buffered saline (PBS) to recover parasite eggs [40]. Three infected livers were dissected, finely sliced, and homogenised in 45 mL PBS containing 200 µL collagenase β (20 mg in 200 µL Milli-Q water (Millipore, Mississauga, ON, Canada)) and 450 µL of 1% penicillin/streptomycin. The mixture was sealed with parafilm and incubated on a rocker at 150 rpm at 37 °C for 16 h to facilitate tissue digestion.

The suspension was centrifuged at 400× *g* for 5 min at room temperature, and the supernatant was removed. The pellet was washed three times with 50 mL PBS, with centrifugation repeated at 400× *g* for 5 min between washes. The mixture was then resuspended in 25 mL PBS and passed sequentially through 250 µm and 150 µm cell strainers to remove tissue debris.

Following centrifugation at 400× *g* for 5 min, the pellet was resuspended in 15 mL PBS. Aliquots (5 mL) were layered onto Percoll gradients (8 mL Percoll in 32 mL 0.25 M sucrose in PBS) in 50 mL tubes and centrifuged at 800× *g* for 5 min. The lower fractions containing eggs were collected, combined, washed twice with PBS, and centrifuged at 400× *g* for 5 min.

Eggs were resuspended in pH-neutral Milli-Q water and exposed to light for 2 h to stimulate miracidial hatching. Miracidia were collected from the upper layer, pooled, and concentrated by centrifugation at 5000× *g* for 15 min. The pellet was homogenised in acidified methanol (methanol/acetic acid/Milli-Q water, 90:9:1, *v*/*v*/*v*), centrifuged at 12,000× *g* for 15 min, and the supernatant was collected and stored at −80 °C prior to lyophilisation using a Savant SpeedVac concentrator (Thermo Scientific, Waltham, MA, USA).

### 4.3. uHPLC Tandem QTOF MS/MS Analyses

Lyophilised samples were resuspended in 35 µL of 0.1% formic acid in Milli-Q water, and 15 µL were analysed by LC–MS/MS using an ExionLC liquid chromatography system (AB SCIEX, Concord, ON, Canada) coupled to a QTOF X500R mass spectrometer (AB SCIEX, Concord, ON, Canada) equipped with an electrospray ionisation source, as described previously [41]. A 25 µL aliquot was injected onto a 100 mm × 1.7 µm Aeris PEPTIDE XB-C18 uHPLC column (Phenomenex, Sydney, Australia) fitted with a SecurityGuard column. Solvent A consisted of 0.1% formic acid in Milli-Q water, and solvent B consisted of 0.1% formic acid in 100% acetonitrile. Peptides were separated using a linear gradient of 3–35% solvent B over 10 min at a flow rate of 400 µL/min, followed by gradients from 35 to 80% solvent B over 2 min and from 80 to 95% solvent B over 1 min.

The ion spray voltage was set to 5500 V, with a declustering potential of 100 V. Curtain gas was set at 30, ion source gas 1 at 40, ion source gas 2 at 50, and the source temperature at 450 °C. Data were acquired in information-dependent acquisition (IDA) mode. Full-scan TOF-MS spectra were collected over a mass range of *m*/*z* 350–1400, and product ion MS/MS spectra were acquired over *m*/*z* 50–1800. Precursor ions exceeding a threshold of 100 counts per second (cps) with charge states of +2 to +5 were selected for fragmentation. Data acquisition and processing were performed using SCIEX OS software version 2.0 (AB SCIEX, Concord, ON, Canada).

### 4.4. Protein Identification

LC–MS/MS raw data were converted using the MSConvert module of ProteoWizard (v3.0.1) [42] and imported into PEAKS Studio (Bioinformatics Solutions Inc., Waterloo, ON, Canada; v7.0) for analysis. The *Schistosoma mansoni* protein database downloaded from WormBase ParaSite (https://parasite.wormbase.org/Schistosoma_mansoni_prjea36577/Info/Index, accessed on 1 September 2021) was used for peptide identification. Raw spectra were analysed using de novo sequencing, database searching, and identification of specific post-translational modifications (PTMs). The false discovery rate (FDR) was set to ≤1%, and scores were reported as −10log(*p*), where *p* represents the probability that an observed match occurred by random chance. The PEAKS search parameters were as follows: (i) precursor ion mass tolerance of 20 ppm; (ii) fragment ion mass tolerance of 0.1 Da; (iii) no enzyme specificity selected; and (iv) monoisotopic precursor and fragment ion masses. Variable modifications included N-terminal acetylation, deamidation of asparagine and glutamine, oxidation of methionine, conversion of glutamic acid and glutamine to pyroglutamate, and C-terminal amidation.

### 4.5. Prediction of Putative Neuropeptides, Gene Ontology, and KEGG Pathway Analysis

Identified proteins were subjected to BLASTp searches against the non-redundant protein sequence database of the National Centre for Biotechnology Information (NCBI). N-terminal signal peptides were predicted using SignalP 4.1 [43] and PrediSi [44] (http://www.predisi.de, accessed on 13 October 2021), with the transmembrane (TM) domains predicted by TMHMM 2.0 [45]. For SignalP analysis, positive signal peptide predictions were accepted only when both the neural network and hidden Markov model algorithms produced concordant results. A D-cutoff value of 0.34 was applied for both SignalP-noTM and TM networks to increase prediction sensitivity.

Putative neuropeptides were predicted using NeuroPred version 1.0 [46], which was used to identify potential peptide cleavage sites within precursor proteins. In addition, peptides identified exclusively by de novo sequencing from LC–MS/MS data that showed sequence similarity to known neuropeptides were also considered as putative neuropeptides. However, these candidates were not included in subsequent behavioural assays.

### 4.6. Protein–Protein Interaction (PPI) Network

Protein–protein interaction (PPI) networks were analysed following a procedure similar to that described previously [10]. Briefly, interactions between annotated *S. mansoni* proteins identified in this study and the complete *S. mansoni* proteome were predicted using STRING version 11.4 [47]. The STRING database integrates protein interaction information from multiple sources, including both direct (physical) and indirect (functional) associations. As physical interactions have not been experimentally established for these proteins and could not be confirmed in this study, our analysis focused on indirect associations, including co-expression relationships and predicted domain–domain interactions.

All available evidence sources within STRING were used to construct the network, and “confidence” was selected as the network edge parameter. To increase network sensitivity, the minimum combined interaction score was set to 0.15. Co-expression interactions were defined as genes showing similar mRNA expression patterns based on previous transcriptomic studies. Proteins without predicted interactions were excluded from the final network. Topological and statistical analyses of the PPI network were performed using the NetworkAnalyzer plugin (https://apps.cytoscape.org/apps/networkanalyzer, accessed on 1 September 2021) in Cytoscape v3.7.1 [48], and the final network was visualised using Cytoscape [48].

### 4.7. Comparative Sequence Analysis of Putative Neuropeptides

Putative *Schistosoma mansoni* neuropeptides were annotated by BLASTp (v2.10.0) searches against the non-redundant protein database of the National Centre for Biotechnology Information (NCBI; accessed on 21 May 2020). Homologous sequences matching the predicted *S. mansoni* neuropeptides with an e-value ≤ 0.5 were retrieved and used for multiple sequence alignment. To reduce low-confidence matches, only sequences with ≥50% identity to the putative neuropeptide precursor proteins were retained.

Multiple sequence alignments were generated using MEGA X (v10.1.8) [49] with the ClustalW algorithm (gap opening penalty: 10; gap extension penalty: 0.2). Alignments were visualised using TeXworks software (v.0.6.5) [50] and edited in Microsoft PowerPoint. Phylogenetic analysis was performed using the Maximum Likelihood method based on the Jones–Taylor–Thornton (JTT) substitution model. The tree with the highest log-likelihood value was selected [51]. Initial trees for heuristic searches were generated automatically using Maximum Likelihood and BioNJ algorithms based on pairwise distances estimated under the JTT model, and the topology with the highest log likelihood was retained. Branch lengths are shown as the number of substitutions per site. Positions containing gaps or missing data were excluded from the analysis.

### 4.8. Peptide Synthesis and Preparation

Neuropeptide precursor proteins identified from *S. mansoni* miracidia were prioritised based on several criteria. Candidate precursors were required to be *Schistosoma*-specific according to BLASTp searches against the NCBI protein database and to be expressed in the miracidia stage [28]. In addition, peptides were required to be relatively small (<2 kDa) to increase the likelihood of interaction with the parasite during behavioural assays. Based on these criteria, eight peptides were selected for behavioural testing (Table 1) and synthesised by ChinaPeptides Co., Ltd. (Shanghai, China). Four peptides were derived from precursor proteins newly identified in this study (Smp_176700 and Smp_319950), while five peptides originated from previously characterised neuropeptide precursors (Smp_004710, Smp_142160, and Smp_201600) [16,17]. Three peptides (NP6, NP7, and NP8) had also been examined previously in behavioural assays with *S. mansoni* cercariae [8]. Among the selected peptides, five were predicted solely by NeuroPred (NP6, NP7, NP10, NP12, and NP13), whereas three were additionally supported by MS/MS spectra (NP8, NP11, and NP14). All peptides were synthesised at ≥95% purity as determined by reverse-phase HPLC and dissolved in Milli-Q water to a final concentration of 0.01 mg/mL for use in both acute and prolonged exposure behavioural assays.

### 4.9. Schistosoma mansoni Miracidia Behavioural Bioassay

Swiss mice infected with *Schistosoma mansoni* were euthanised using CO_2_ inhalation, and their livers were perfused with room-temperature phosphate-buffered saline (PBS) to recover parasite eggs. Two infected mouse livers were dissected, finely sliced, and homogenised in 50 mL PBS. The suspension was centrifuged at 2000× *g* for 10 s at 20 °C, and the supernatant was removed. The pellet was resuspended in 50 mL PBS and centrifuged again. This washing step was repeated three times until the supernatant became clear.

The eggs were then distributed into three 50 mL tubes containing pH-neutral water. The lower portion of each tube was wrapped in aluminium foil, while the upper portion was exposed to light for 2 h at room temperature to stimulate miracidial hatching. Every 30 min, the upper 4 mL of water was collected, and the number of miracidia was estimated by examining 100 µL aliquots under a microscope. Miracidia were concentrated by centrifugation at 4000× *g* for 15 min at 20 °C, and the supernatant was removed. The pellet was resuspended in 10 mL Milli-Q water and vortexed to obtain a suspension containing approximately 30 miracidia per 100 µL.

Behavioural bioassays were performed as described previously [11]. Briefly, 100 µL aliquots of miracidia were placed on hydrophilic glass slides (StarFrost^®^ Superclean, ProSciTech Pty Ltd., Kirwan, Australia) and observed using an Olympus CKX41 microscope (Olympus, Tokyo, Japan) equipped with an Olympus DP22 digital camera (15 frames s^−1^, 2.8 MP resolution) (Olympus, Tokyo, Japan). Miracidial behaviour was recorded for 1 min prior to treatment, followed by the addition of 2 µL of neuropeptide solution and a further 1 min of recording. Each neuropeptide treatment was tested in nine replicates. Negative control data (Milli-Q water) were obtained from recordings generated using identical methods in a previous study [10].

Video analysis was conducted as described previously [11]. Videos were divided into pre- and post-treatment segments and analysed using FIJI software (Version 1.53c, National Institute of Health (NIH), Bethesda, MA, USA) [52]. Background subtraction using a rolling mean method enhanced the contrast of miracidia [53]. The TrackMate plugin version 7 [54] was used to track miracidial positions in each frame and assemble movement trajectories within the field of view. Movement parameters were calculated using the MTrackJ plugin version 1.5.1 [55], including average velocity (mm s^−1^), angular standard deviation (degrees), duration of presence within the field of view (s), and the number of tracks per minute.

Statistical analyses were performed in R version 4.1.0 using an aligned-rank transform (ART) implemented in the ARTool package (v0.10.0) to conduct a nonparametric two-way ANOVA, with neuropeptide treatment as a between-subjects factor and pre/post addition as a within-subjects factor [56]. Post hoc contrasts compared behavioural changes before and after neuropeptide addition against the Milli-Q water control [57]. Differences were considered significant at *p* < 0.05. Statistical analysis and figure generation were performed in RStudiov version 4.1.0 [58] using multiple packages, including readxl v1.3.1, tidyverse v1.3.1, magrittr v2.0.1, forcats v0.5.1, lme4 v 1.1-27.1, AICcmodavg v2.3, car v 3.0-12, multcomp v1.4-16, ggplot2 v3.3.3, plotrix v3.8-1, ARTool v0.10.0, and UpSetR v1.4.0 [56,59,60,61,62,63,64,65,66,67,68,69]. In boxplots, points beyond 1.5× the interquartile range above the upper quartile or below the lower quartile were defined as outliers.

### 4.10. Long-Term Neuropeptide Exposure

Prolonged behavioural bioassays were conducted over a 360 min period to examine the long-term effects of neuropeptides on miracidial behaviour. Aliquots of 8 µL neuropeptide solution (0.01 mg/mL in Milli-Q water) were added to tubes containing 500 µL of water with miracidia, yielding a final concentration of 157 ng/mL. Control samples received Milli-Q water only. For each tube, 100 µL aliquots were collected prior to neuropeptide addition (pre-exposure) and subsequently every 90 min over the 360 min exposure period. Each aliquot was placed onto a glass slide, and miracidial behaviour was recorded for 1 min using the microscopy setup described above. Nine replicates were performed for each neuropeptide treatment and for the control group. Behavioural parameters obtained for each neuropeptide treatment were compared with the control across the pre-exposure and post-exposure time points. Statistical analyses were conducted as described above, with additional post-exposure time points incorporated into the within-subjects analysis. This experiment was designated as the prolonged exposure assay. Given the low concentration of neuropeptides used (0.01 mg/mL), measurement of pH changes in the solutions was considered unnecessary.

### 4.11. Secondary Structure Analysis

Secondary structures of putative neuropeptides that produced significant effects in behavioural bioassays were predicted using PEP-FOLD3 (https://bioserv.rpbs.univ-paris-diderot.fr/services/PEP-FOLD3/, accessed on 1 October 2021). PEP-FOLD3 performs de novo structure prediction of short peptides using a greedy assembly approach based on Hidden Markov Model-derived structural alphabets [70]. The predicted peptide structures were visualised using VMD v1.9.3 [71].

## Figures and Tables

**Table 1:** List of putative neuropeptides synthesised in this study.

ID	Sequence	Molecular Weight (Da)	Accession of Precursor
NP6	ASLSYF-OH	686.76	Smp_201600.1 (Sm-npp-36)
NP7	FLLALPSP-OH	857.05	Smp_004710.1 (Sm-npp-30)
NP8	NYLWDTRL-NH_2_	1079.21	Smp_142160.1 (Sm-npp-17)
NP10	LLMSVAGLHH-OH	1077.31	Smp_176700.1 (Smp-npp-41)
NP11	RSELSDSPSSLSLSPSSSS-OH	1909.96	Smp_201600.1 (Sm-npp-36)
NP12	LSQPSYSFNRY-OH	1361.46	Smp_004710.1 (Sm-npp-30)
NP13	FLLGLPPSLRQH-OH	1377.64	Smp_004710.1 (Sm-npp-30)
NP14	FSFIFDFLKNILRI-OH	1733.13	Smp_319950.1 (Smp-npp-42)

**Table 2:** Two-way ART ANOVA results, comparing changes in *S. mansoni* miracidia behaviour following exposure to NP6, NP8, NP10, NP11, NP12, and NP13 at 0.01 mg/mL to Milli-Q water pre-exposure and at 90 min intervals over 360 min post-exposure. Test statistics, degrees of freedom, and *p*-values for the main effects of putative neuropeptide and period (pre-exposure vs. 90 min-, 180 min-, 270 min-, and 360 min-post-exposure), and their interaction. If the interaction effect was significant, pairwise contrast tests analysed the significance of the change in behaviour pre vs. post exposure between Milli-Q and each of the putative neuropeptides. Metrics: mean velocity (mm/s), average duration of presence (s), angular SD (degrees), and average number of tracks per min.

Metrics	Effect	Statistic	df	*p*-Value	Interval	NP6	NP8	NP10	NP11	NP12	NP13
Mean velocity (mm/s)	Neuropeptide	7.9375	6	**0.0000**							
	Period	27.6602	4	**0.0000**							
	Neuropeptide: Period	1.9793	24	**0.0061**	0–90	0.0930	0.0893	0.7978	0.3427	0.0742	0.6206
					0–180	0.0648	0.3960	0.6466	0.2949	0.4507	0.4168
					0–270	**0.0022**	0.1586	0.2421	0.8308	0.6161	0.6389
					0–360	**0.0006**	0.3075	0.0896	0.0557	0.4483	**0.0193**
Average duration of presence (s)	Neuropeptide	8.6891	6	**0.0000**							
	Period	16.5836	4	**0.0000**							
	Neuropeptide: Period	3.5646	24	**0.0000**	0–90	0.5300	0.4477	0.2993	0.9553	0.1968	0.8618
					0–180	0.6772	0.8012	0.4670	0.4012	**0.0484**	0.3750
					0–270	**0.0238**	0.3357	0.9297	0.5779	0.0813	0.6506
					0–360	**0.0009**	0.3602	0.0932	0.7706	0.1102	**0.0008**
Angular SD (degrees)	Neuropeptide	6.6137	6	**0.0000**							
	Period	8.4617	4	**0.0000**							
	Neuropeptide: Period	1.8949	24	**0.0096**	0–90	0.2398	0.7700	0.6453	0.9596	0.8274	0.5170
					0–180	0.4498	0.6318	0.6987	0.8357	0.9529	0.8390
					0–270	0.0890	0.2018	0.0869	0.5957	0.9224	0.1928
					0–360	**0.0071**	0.1288	0.1269	0.1620	0.8070	**0.0389**
Tracks per min	Neuropeptide	20.8075	6	**0.0000**							
	Period	41.6147	4	**0.0000**							
	Neuropeptide: Period	2.7628	24	**0.0000**	0–90	0.3716	0.4263	0.8251	**0.0053**	0.2689	0.1405
					0–180	**0.0276**	**0.0023**	0.4204	**0.0129**	**0.0183**	0.0602
					0–270	**0.0094**	0.0504	0.4075	**0.0000**	**0.0247**	**0.0010**
					0–360	0.3366	0.3048	**0.0252**	0.3010	0.1725	0.3607

## Data Availability

The original contributions presented in this study are included in the article/Supplementary Materials. Further inquiries can be directed to the corresponding author.

## Supplementary Materials

The following supporting information can be downloaded at: https://www.mdpi.com/article/10.3390/ijms27062839/s1.
