# Supplementary material for: Peptidomic Identification of Behaviour-Modulating Putative Neuropeptides in Schistosoma mansoni Miracidia
Source: Int J Mol Sci. 2026 Mar 20;27(6):2839. doi: 10.3390/ijms27062839 (PMC13026224; doi:10.3390/ijms27062839)
Supplement: Supplementary file 1 [file ijms-27-02839-s001.zip › Supplementary materials.pdf]

Supplementary Information

File S1: A summary of putative neuropeptide precursor proteins identified in this study. Peptides supported by LC-MS are bold and underlined, all putative neuropeptides employed in behavioural bioassays were highlighted in blue and all other putative neuropeptides were highlighted in yellow. The physical properties of each putative neuropeptide, including percentage of hydrophobic, acidic, basic and neutral regions, are also included. Phylogenetic analysis of all putative neuropeptide precursors against the NCBI database identified using BLASTp. All matches with at least 50% identity were included. Colour: Red: *Schistosoma*; Blue: *Heterobilharzia*; Green: *Trichobilharzia*; Bold: Putative neuropeptide precursor.

Figure S1: Statistical analysis of PPIs shown in **Figure 2A**. **(A)** Frequencies of path lengths; **(B)** the degree values of the nodes; **(C)** the relationships between number of neighbours and betweenness centrality and **(D)** closeness centrality; **Figure S2**: Changes in *S. mansoni* miracidial behaviour from one min pre-exposure and post-exposure to Milli-Q water and 3 mg/mL NP6, NP8, NP10, NP11, NP12, NP13. **(A)** Average velocity (mm/s); **(B)** Angular SD (degrees); **(C)** Duration of presence (s); **(D)** miracidia per min. Boxplot indicates median, 25th and 75th percentiles, minimum and maximum data with outliers represented by dots. A two-way ART-ANOVA test was used to calculate P values for the mixed-effects interaction of pre-exposure and post-exposure neuropeptide treatments against Milli-Q water: $*P < 0.05$, $**P < 0.01$ and $***P < 0.001$.

Figure S3: Changes in *S. mansoni* miracidial behaviour from one min pre-exposure and post-exposure to Milli-Q water and 0.1 mg/mL NP6, NP8, NP10, NP11, NP12, NP13. **(A)** Average velocity (mm/s); **(B)** Angular SD (degrees); **(C)** Duration of presence (s); **(D)** miracidia per min. Boxplot indicates median, 25th and 75th percentiles, minimum and maximum data with outliers represented by dots. A two-way ART-ANOVA test was used to calculate P values for the mixed-effects interaction of pre-exposure and post-exposure neuropeptide treatments against Milli-Q water.

Figure S4: Structure of bioactive peptides and comparative sequence analysis of their respective precursor proteins, including information of conserved amino acids. **(A)** Sm-npp-30 and NP7 (FLLALPSP-OH) and NP12 (LSQPSYSFNRY-OH); **(B)** Sm-npp-17 and NP8 (NYLWDTRL-NH_2); **(C)** Smp-npp-41 and NP10 (LLMSVAGLHH-OH) and **(D)** Sm-npp-36 and NP11 (RSELSDSPSSLSLSPSSSS-OH). Structural Alphabet (SA) (vertical axis) at each position. Colour code: red: helical, green: extended, blue: coil. Heatmaps and structural models were generated using PEP-FOLD3.

Table S1: Protein identification, supporting peptides, annotations (incl. SignalP and TMHMM results) and de novo only peptide sequences (average local confidence>80%); **Table S2**: List of putative neuropeptide precursor proteins identified, including annotations, lowest e-value, putative neuropeptide sequences, genome location and species specificity. Colour codes: yellow-sequences supported by MS/MS spectra; orange-sequences partially supported by MS/MS spectra, the segments with MS/MS spectra shown in parentheses.

Table S3: The protein-protein interactions of identified proteins. Putative neuropeptide precursor proteins are denoted with an asterisk (*).

Table S4: The relative gene expression of all identified proteins, including precursors of putative neuropeptides (values are transcripts per million units, TPMs) at different stages: miracidia (M), sporocyst (S) and cercariae (C) and mature schistosome (Ma).

Table S5: Behavioural bioassay guided data, including track and points data from *S. mansoni* miracidia one min pre-exposure and post-exposure to Milli-Q water and all 6 neuropeptides at 3, 0.1, and 0.01 mg/mL, including NP6, NP8, NP10, NP11, NP12 and NP13.

Table S6: Statistical analysis of acute (1-min) *Schistosoma mansoni* miracidial behavioural responses following exposure to putative neuropeptides (NP6, NP8, NP10, NP11, NP12 and NP13) at 3, 0.1, and 0.01 mg/mL.

Table S7: Behavioural bioassay guided data, including track and points data from *S. mansoni* miracidia pre-exposure and 90 min, 180 min, 270 min and 360 min post-exposure to Milli-Q water and all 6 neuropeptides at 0.01 mg/mL, including NP6, NP8, NP10, NP11, NP12 and NP13.

Movie S1: A representative video of *S. mansoni* miracidia bioassay 360 min post-exposure using a 0.01 mg/mL dilution of Milli-Q water (Speed × 2).

Movie S2: A representative video of *S. mansoni* miracidia bioassay 360 min post-exposure using a 0.01 mg/mL dilution of NP6 (Speed × 2).

Movie S3: A representative video of *S. mansoni* miracidia bioassay 360 min post-exposure using a 0.01 mg/mL dilution of NP13 (Speed × 2).
