# Supplementary material for: Peptidomic Identification of Behaviour-Modulating Putative Neuropeptides in Schistosoma mansoni Miracidia
Source: Int J Mol Sci. 2026 Mar 20;27(6):2839. doi: 10.3390/ijms27062839 (PMC13026224; doi:10.3390/ijms27062839)

Characterization of neuropeptides in *S. mansoni* miracidia

Smp_142160.1_Sm-npp-17

MFHHYVLVLICFIAFTSVQHIDATDDNFASSKEQSTKFAPIRRHMMKR**NYLWDTRLG**KRSMNREAFPWYNHDLYNYYNDDNFRGYYTGY
DD

NP8: Hydrophobic: 37.5% Acidic: 12.5% Basic: 12.5% Neutral: 37.5%

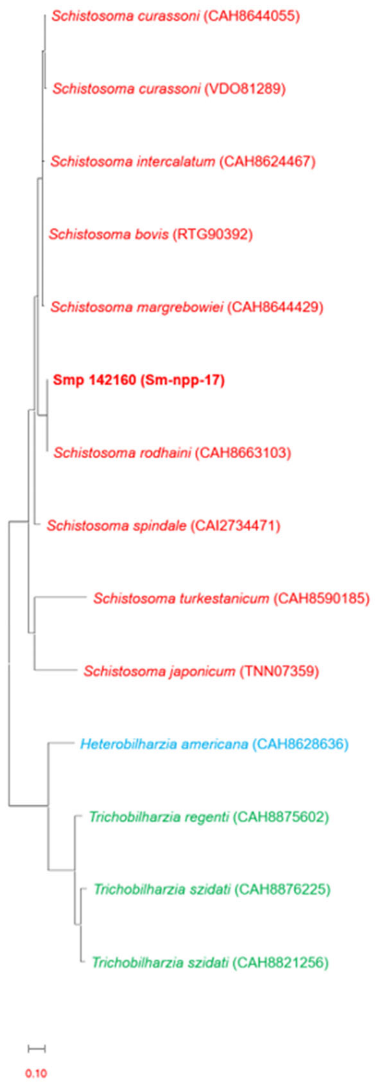

Smp_004710.1_Sm-npp-30

MTNYWFQLFCCMIIGFLILTSHTNCMDSDNDPSDISADKRFLLALPSPKRLSQPSYSFNRYRRPMYGYGYRPDYIDADDDLFNEDKRFLLGL PPKVEHKRFLLGLPPSLRQHKR**FILGLPAPTRFHS**

NP7: Hydrophobic: 87.5% Acidic: 0% Basic: 0% Neutral: 12.5%

NP12: Hydrophobic: 27.27 % Acidic: 0% Basic: 9.09% Neutral: 63.64%

NP13: Hydrophobic: 58.33% Acidic: 0% Basic: 16.67% Neutral: 25%

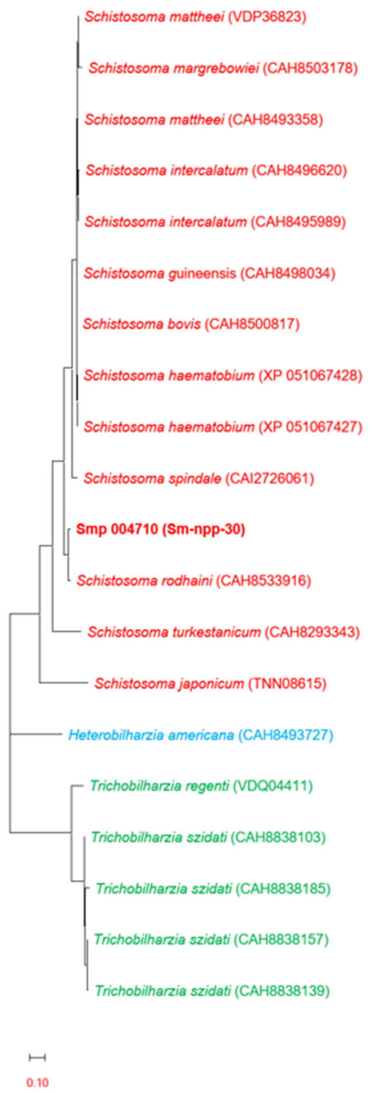

Smp_201600.1_Sm-npp-36

MNKLSLIQLFIIILVTLTIKINGDNTDTYDKRASLAYFKR**RSELSDSPSSLSLSPSSSS**KRASLSYFKRGQYDQRFEGIHHIRQIIEICPCLNKDYLHWLEENLLLNKHTTNTNDDDNLELEKRASLSYF

NP6 Hydrophobic: 50% Acidic: 0% Basic: 0% Neutral: 50%

NP11: Hydrophobic: 26.32% Acidic: 10.53% Basic: 5.26% Neutral: 57.89%

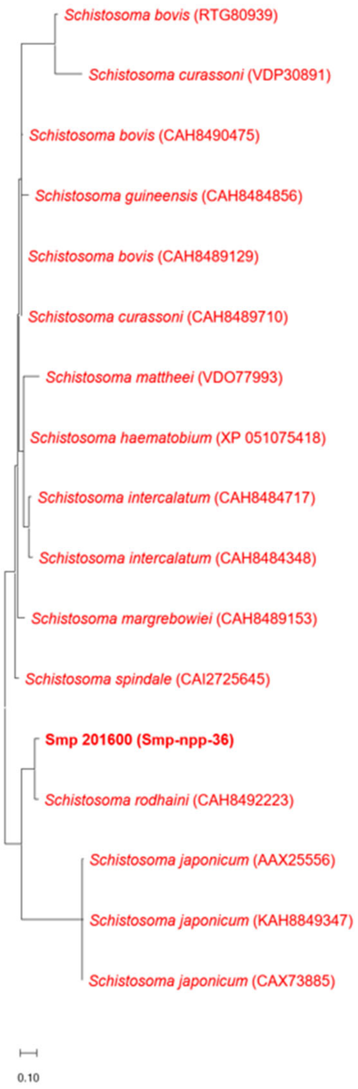

Smp_200440.1_Sm-npp-40

MHKYYHYNLCTSKKHLFILTTYVICTILCINSESLDDSSLLIDFPCGPHPIDMFLSKLFDYLEKQETPIMDTGETDEKLHIKMIYGKLLYAFTPMP
RSSYYVYNEIMRSINGWNECMSKQPRLKKWLKRR**NFLQNVNTIVRPNLNTDKSTY**KH

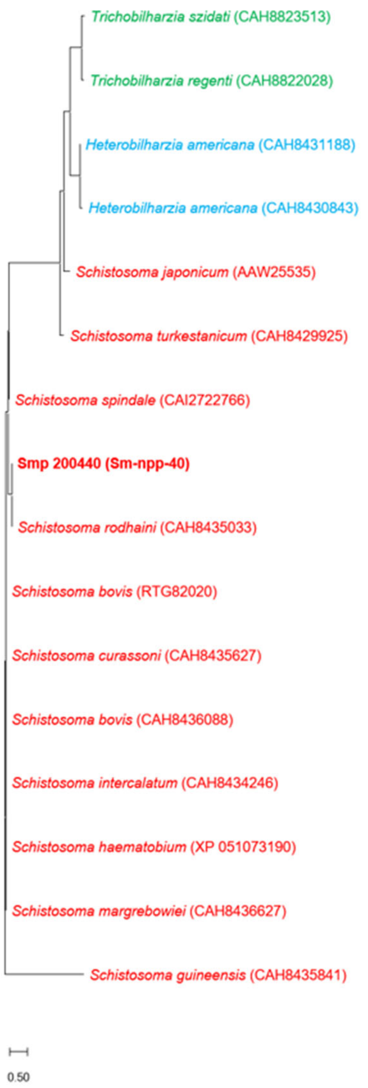

Smp_176700.1_ Sm-npp-41

MKITIVNVTILVLVILSVMSLGPHCLLMSVAGLHHKR**SISVDVVEAEPMPELDDANGYGLPNQID**

NP10: Hydrophobic: 60% Acidic: 0% Basic: 20% Neutral: 20%

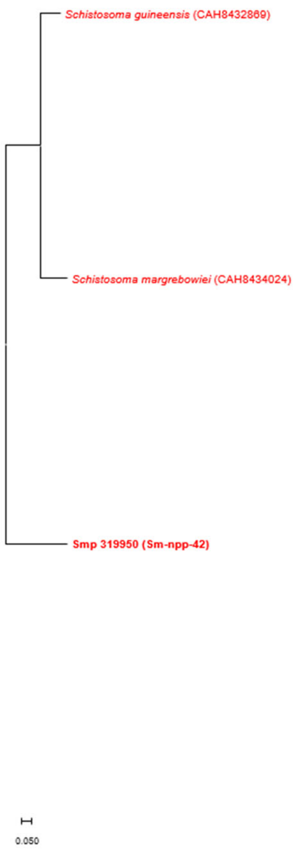

Smp_319950.1_ Sm-npp-42-form A

MHLTFIVTFTVLILVNFPNVNCLLKLDDAEKERLKDDFVKEINEELKKLPPSEHSIFSEPRQKR**FSFIFDFLKNILRI**

NP14: Hydrophobic: 64.29% Acidic: 7.14% Basic: 14.29% Neutral: 14.29%

Smp_320030.1_Sm-npp-42-form B

MHLTFIVTFTVLILVNFPNVNCLLKLDDAEKERLKDDFVKAINEELKKLSPGDQSISSERRKKH**GFGESILGLASMYG**K

Smp_320000.1_ Sm-npp-42-form C_1aa different from form-B

MHLTFIVTFTVLILVNFPNVNCLLKLDDAEKERLKDDFVKAINEELKKLSPGDQSISSERRKKR**GFGESILGLASMYG**K

Smp_321760.1_ Sm-npp-44

MISLLLFSNLLIISIQG**VVIPEANPIDYQS**KRWTDFKRAIRWSEYPFTFTPEIKRHYLYEQRPYYNDILV

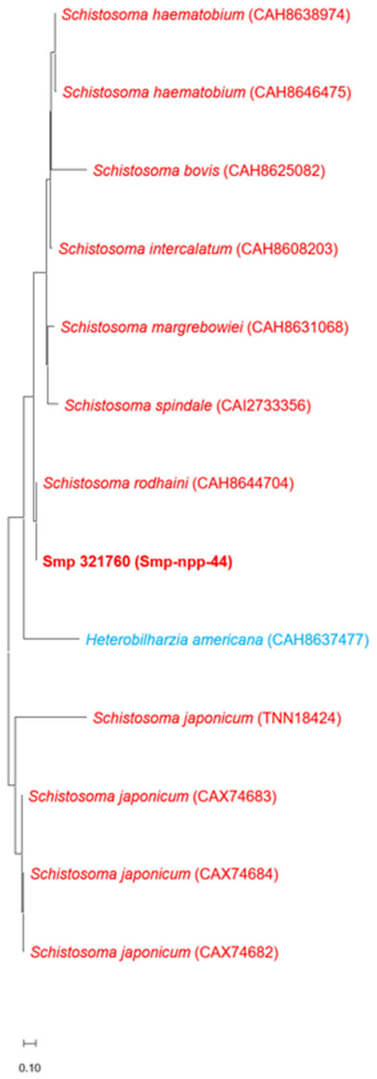

Smp_346020.1_ Sm-npp-45

MKIYIFVFVCIVLLQSVIG**KHDQQVVQESHDGHLVYPQNFQ**KRSFINKQLQIILVYDENLKQNEMFPKIQKAMNSASSYWEKTLKVKLNSK
KAIHFPRECTDKSHVVEVNNNIRSCVNAQCKEDTEIEGVKIPNKYLSSCHHQMNNEYKEIYSEGSGLAPNQLLIVVTGPCKIPSYESIHISGVIV
RTYPSTER

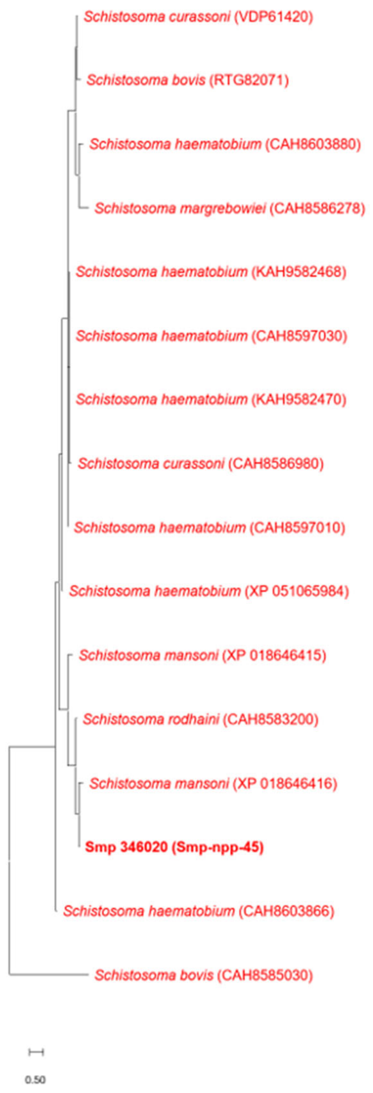

Supplement: Supplementary file 1 [file ijms-27-02839-s001.zip › File S1.pdf]
