# Supplementary figures and images for: Peptidomic Identification of Behaviour-Modulating Putative Neuropeptides in Schistosoma mansoni Miracidia
Source: Int J Mol Sci. 2026 Mar 20;27(6):2839. doi: 10.3390/ijms27062839 (PMC13026224; doi:10.3390/ijms27062839)

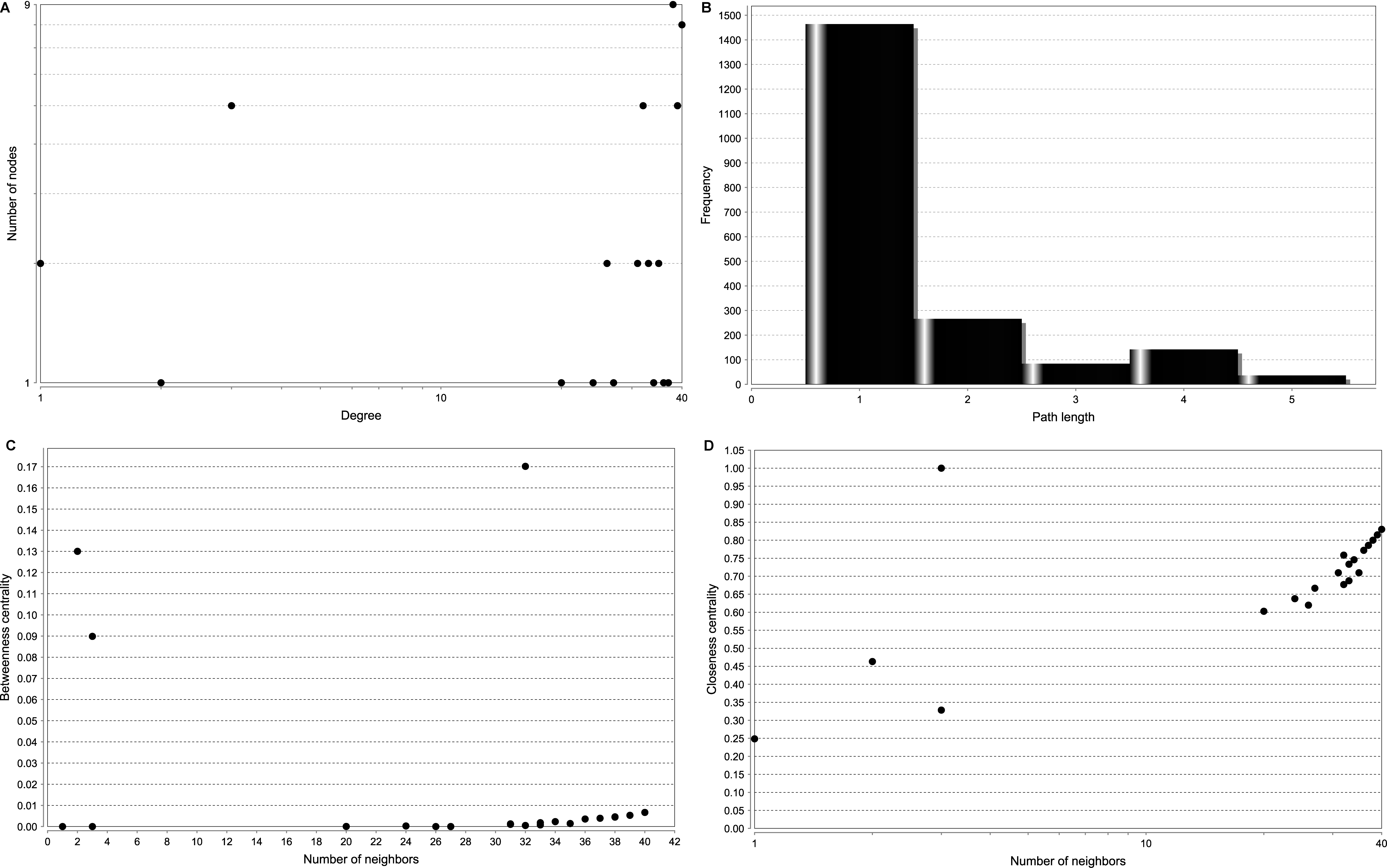

Supplement: Supplementary file 1 [file ijms-27-02839-s001.zip › Figure S1.tif]

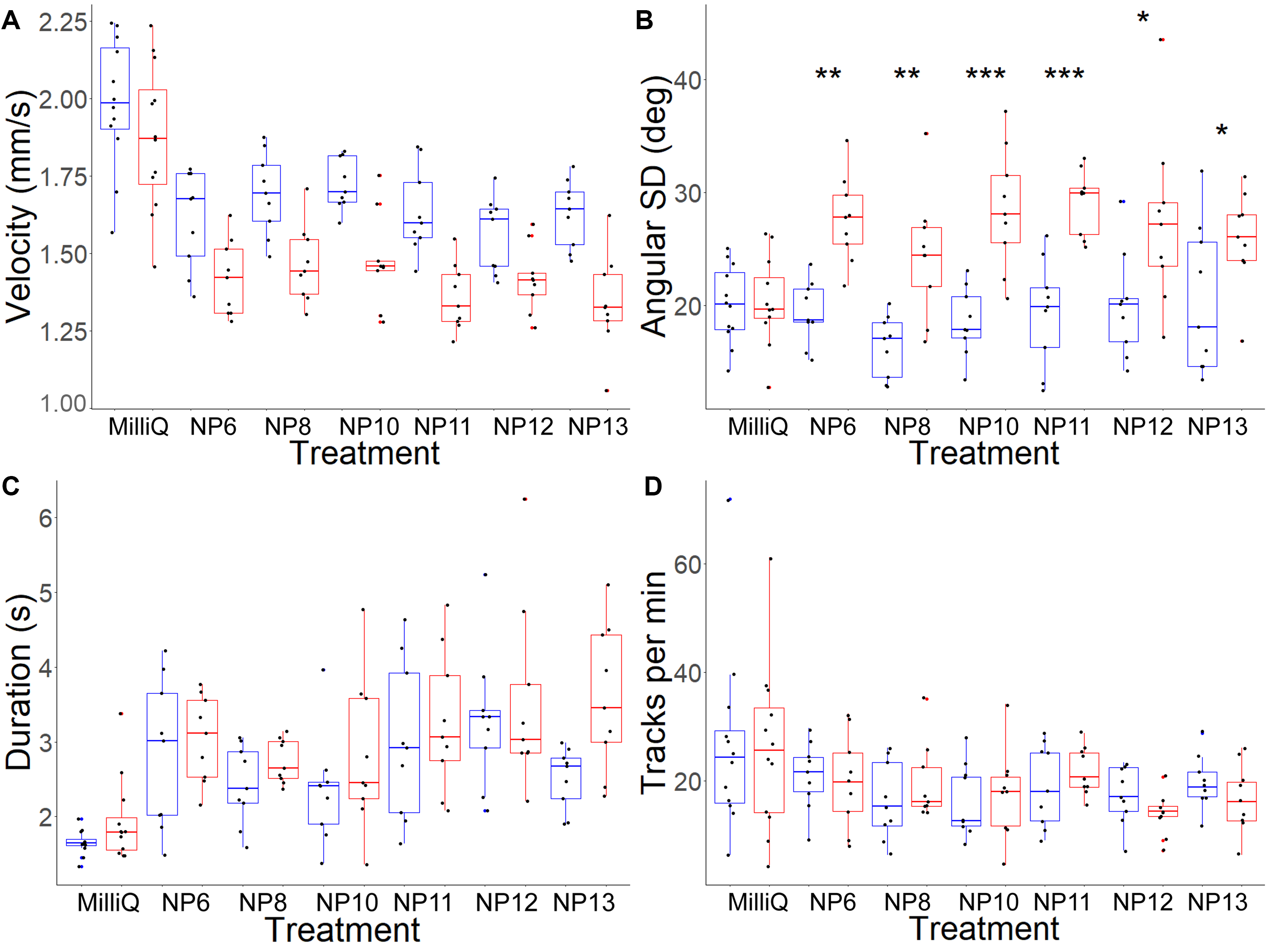

Supplement: Supplementary file 1 [file ijms-27-02839-s001.zip › Figure S2.tif]

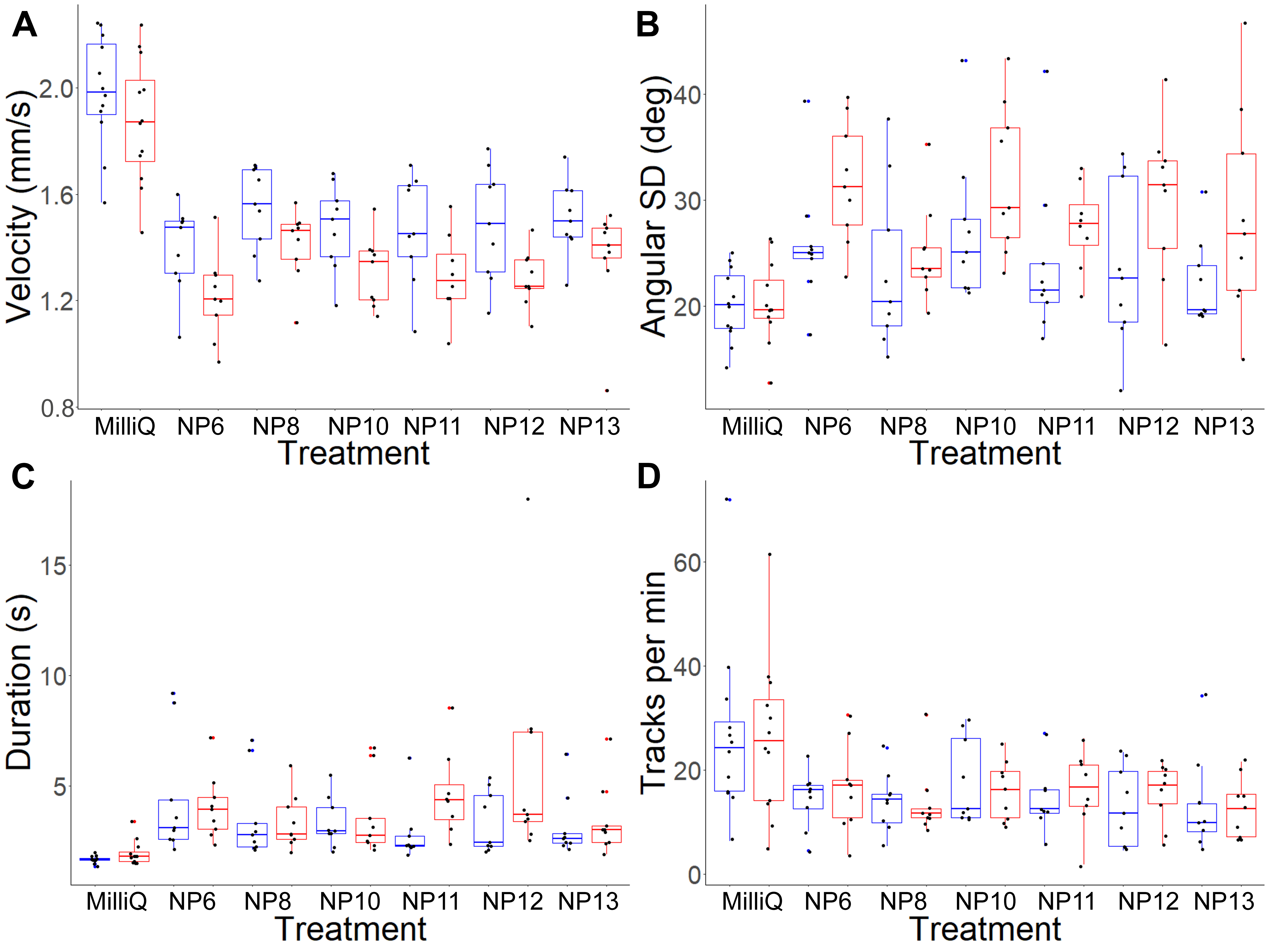

Supplement: Supplementary file 1 [file ijms-27-02839-s001.zip › Figure S3.tif]

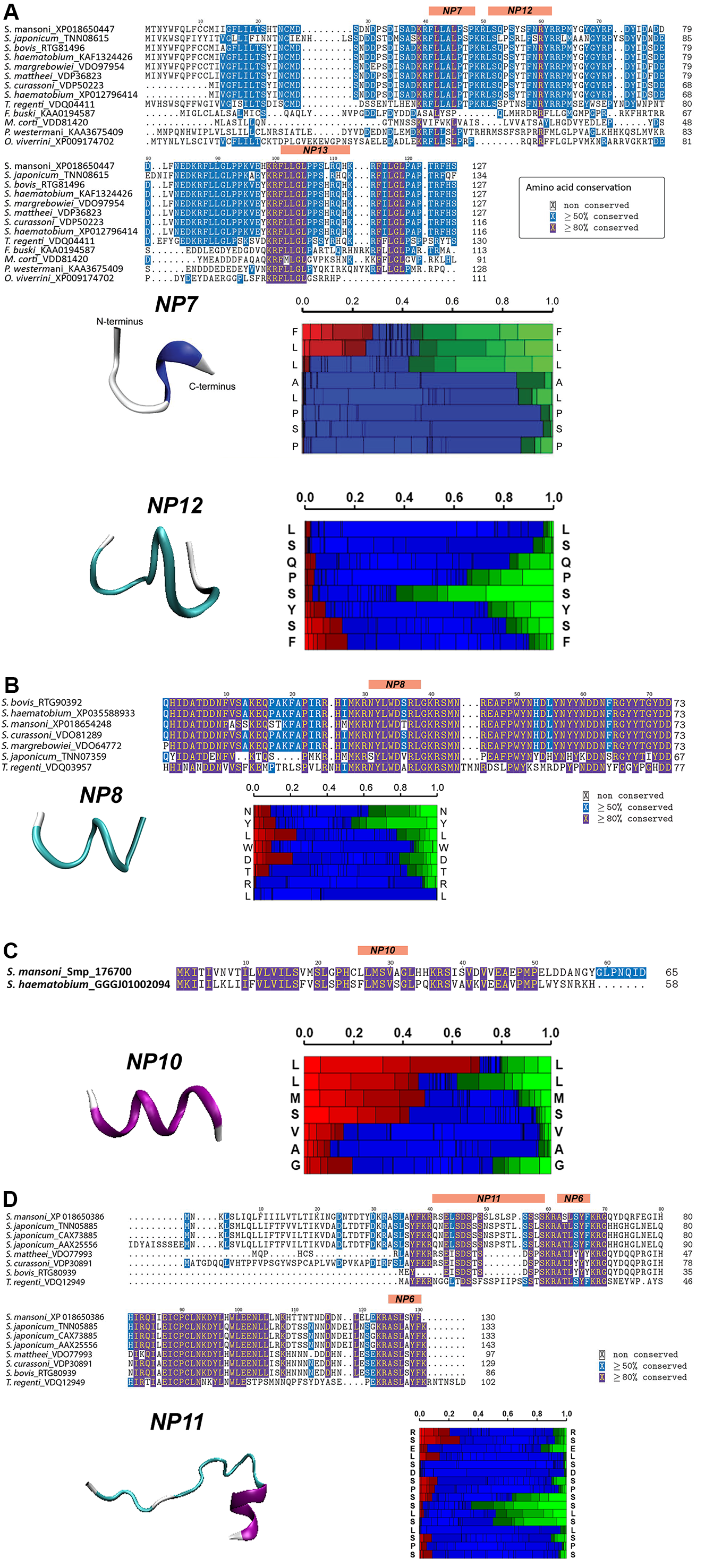

Supplement: Supplementary file 1 [file ijms-27-02839-s001.zip › Figure S4.tif]
